# Immune response characterization in a human challenge study with a *Shigella flexneri* 2a bioconjugate vaccine

**DOI:** 10.1016/j.ebiom.2021.103308

**Published:** 2021-04-01

**Authors:** Kristen A. Clarkson, Kawsar R. Talaat, Cristina Alaimo, Patricia Martin, A. Louis Bourgeois, Anita Dreyer, Chad K. Porter, Subhra Chakraborty, Jessica Brubaker, Daniel Elwood, Rahel Frölich, Barbara DeNearing, Hailey P. Weerts, Brittany Feijoo, Jane Halpern, David Sack, Mark S. Riddle, Veronica Gambillara Fonck, Robert W. Kaminski

**Affiliations:** a Department of Enteric Infections, Bacterial Diseases Branch, Walter Reed Army Institute of Research, Silver Spring, MD, United States; b Department of International Health, Johns Hopkins Bloomberg School of Public Health, Baltimore, MD, United States; c LimmaTech Biologics AG, Schlieren, Switzerland; d PATH, Washington, DC, United States; e Naval Medical Research Center, Silver Spring, MD, United States

**Keywords:** *Shigella*, Immunogenicity, Bioconjugate vaccine, Human challenge, Gut-homing responses, Parenteral immunization

## Abstract

**Background:**

Diarrheal diseases are a leading cause of global morbidity and mortality affecting all ages, but especially children under the age of five in resource-limited settings. *Shigella* is a leading contributor to diarrheal diseases caused by bacterial pathogens and is considered a significant antimicrobial resistance threat. While improvements in hygiene, and access to clean water help as control measures, vaccination remains one of the most viable options for significantly reducing morbidity and mortality.

**Methods:**

Flexyn2a is a bioconjugate vaccine manufactured using novel conjugation methodologies enzymatically linking the O-polysaccharide of *S. flexneri* 2a to exotoxin A of *Pseudomonas aeruginosa*. The protective capacity of Flexyn2a was assessed in a controlled human infection model after two intramuscular immunizations. Immune responses pre- and post-immunization and/or infection were investigated and are described here.

**Findings:**

Flexyn2a induced lipopolysaccharide (LPS)-specific serum IgG responses post-immunization which were associated with protection against shigellosis. Additionally, several other immune parameters, including memory B cell responses, bactericidal antibodies and serum IgA, were also elevated in vaccinees protected against shigellosis. Immunization with Flexyn2a also induced gut-homing, LPS-specific IgG and IgA secreting B cells, indicating the vaccine induced immune effectors functioning at the site of intestinal infection.

**Interpretation:**

Collectively, the results of these immunological investigations provide insights into protective immune mechanisms post-immunization with Flexyn2a which can be used to further guide vaccine development and may have applicability to the larger *Shigella* vaccine field.

**Funding:**

Funding for this study was provided through a Wellcome Trust grant.

**Research in Context**

**Evidence before this study**

Currently, there are no licensed vaccines to prevent infection with *Shigella* species. A PubMed search of “(*shigella*) AND conjugate vaccine”, restricted to clinical trials, returned 11 articles documenting a history of robust immunogenicity and safety provided by previously investigated *Shigella* conjugate vaccines. While other *Shigella* conjugate vaccines are under clinical investigations, Flexyn2a is the only bioconjugate to undergo efficacy assessments. Furthermore, while an unrestricted PubMed search of “(shigella) AND correlate” returns 73 articles associated with the investigation of immune correlates of protection for *Shigella* infection, a defined mechanistic correlate of protection has yet to be established. The current challenge study was designed to assess the efficacy of the Flexyn2a vaccine, which has an excellent safety and immunogenicity profile as previously published; however, this study also provided the unique opportunity to thoroughly investigate the immune responses associated with protection from *Shigella* infection after parenteral immunization with a conjugate vaccine.

**Added value of this study**

This is the first study to provide a thorough characterization of the systemic and mucosal immune responses induced after parenteral immunization with a *Shigella* conjugate vaccine, as well as to investigate the association of each immune parameter with protection from shigellosis caused by *S. flexneri* 2a. Additionally, this is the first study to report immune response data using the recently published consensus shigellosis endpoint, an outcome to be used in future *Shigella* challenge models and vaccine efficacy studies.

**Implications of all the available evidence**

The current study provides essential insights into protective immune mechanisms associated with parenteral immunization with a *Shigella* bioconjugate. The precedent set in the current study of thorough characterization of the immune responses post-immunization may influence the design and immunological analyses of future *Shigella* clinical studies and can help move the field towards the establishment of an immune correlate of protection for shigellosis.

Alt-text: Unlabelled box

## 1 Introduction

*Shigella* species are one of the leading causes of diarrhea-associated morbidity and mortality across all age groups and is the second leading cause of death in children under the age of 5 years [1,2]. The impact of *Shigella* infection goes beyond the acute illness observed, as children with repeated enteric infections are also at risk of physical and cognitive stunting, ultimately leading to a higher risk of mortality due to other infectious diseases [3,4]. While antibiotics are generally effective in the treatment of shigellosis, globally increasing rates of multiple antibiotic-resistance, particularly in Africa and Asia, requires continual emphasis on primary prevention methods such as an efficacious vaccine [5], [6], [7], [8], [9]. Given the challenges of effectively treating *Shigella* with antibiotics, the Wellcome Trust has recently recommended that vaccine development should be accelerated [5]. Although several *Shigella* vaccine approaches have been attempted in recent years, there is currently no widely available licensed vaccine [10,11].

Epidemiological data and challenge/re-challenge studies have shown that prior *Shigella* infection protects from subsequent infection in a serotype-specific manner [12,13]. As *Shigella* serotypes are determined by the structure of their O-polysaccharide (OPS), the lipopolysaccharide (LPS), or OPS alone, is considered to be a key protective antigen. When considering candidate vaccines, OPS conjugates are of particular interest as the strategy has a well-demonstrated safety and efficacy history [14], [15], [16].

Flexyn2a is a bioconjugate vaccine manufactured using novel conjugation methodologies that enzymatically link the OPS of *S. flexneri* 2a to exotoxin A of *Pseudomonas aeruginosa* (EPA) [17,18]. In general, bioconjugates result from a single fermentation/processing step and have a homogeneous composition which simplifies their physicochemical characterization and, consequently, the number of analytical assays required to release the final product. These attributes, along with the requirement of one unique GMP production to produce a single batch, contribute to a product that is simple, reliable and inexpensive to manufacture [19]. Additionally, the *Shigella* bioconjugate vaccines have demonstrated robust safety and immunogenicity profiles in prior Phase 1 clinical assessments [17,18]. More recently, the Flexyn2a bioconjugate vaccine was assessed in a controlled human infection model (CHIM) to evaluate the preliminary efficacy following challenge with *S. flexneri* 2a strain 2457T with promising results, as described in a companion manuscript (Talaat-2020). Furthermore, robust *S. flexneri* 2a LPS-specific serum IgG and IgA responses were reported post-immunization, with serum IgG responses serving as a correlate of protection post-challenge (Talaat-2020).

Efforts have since been underway to further characterize the humoral immune responses induced post-parenteral immunization with the Flexyn2a bioconjugate, as well as post-oral challenge with *S. flexneri* 2a strain 2457T to investigate potential correlates, or surrogates, of protection associated with *Shigella* infection. These analyses could further contribute to *Shigella* vaccine development efforts as well as enhance understanding of the mechanisms of protection associated with a parenterally administered *Shigella* bioconjugate vaccine. Moreover, data from the current challenge trial with the Flexyn2a bioconjugate were recently used to support the development of a consensus shigellosis endpoint and described herein is the first report of immune response analyses in a Phase 2b trial using this consensus definition [20].

## 2 Methods

### 2.1 Study design and efficacy

Trial design, conduct, randomization, blinding/masking, inclusion/exclusion criteria and efficacy data have been described in detail in a companion manuscript (Talaat-2020). Briefly, the trial was a randomized (1:1), double-blind, placebo-controlled study to assess preliminary efficacy post-immunization with Flexyn2a. As the target population for a *Shigella* vaccine is naïve children <2 years of age, efforts were undertaken to enroll immunologically naïve subjects to better represent this target population. Therefore, subjects with serologic evidence of prior exposure to *S. flexneri* 2a, described as a *S. flexneri* 2a LPS-specific serum IgG titre of ≥2500 [18], were excluded from participation, in addition to other *Shigella*-specific exclusion criteria (Talaat-2020). Additionally, the *Shigella*-specific exclusion criteria provided a more homogeneous population across treatment groups by reducing differences in the immune status of enrolled subjects. Increasing the homogeneity of subjects across treatment groups helped ensure the quality of data output post-challenge as differences in *Shigella*-specific immunity could have influenced or biased efficacy results. Subjects were intramuscularly administered either unadjuvanted Flexyn2a (*n* = 34) or placebo (saline, *n* = 33) on study days 0 and 28. Each dose of Flexyn2a contained 10 μg of *S. flexneri* 2a OPS and 50 μg of EPA. One month after the last immunization (day 56), subjects (30 vaccinees, 29 placebo recipients) were orally challenged with approximately 1500–1700 colony forming units of *S. flexneri* 2a strain 2457T. Five days post-challenge (or sooner if clinically warranted), all subjects initiated antibiotic treatment. Subjects were discharged from the inpatient facility after producing two culture negative stool samples.

### 2.2 Ethics

This trial was approved by the Western Institutional Review Board and was conducted in compliance with all federal regulations governing the protection of human volunteers. The study clinical protocol is published on clinicaltrials.gov (registration NCT02646371). All subjects provided written informed consent and were de-identified.

### 2.3 Blood processing

Whole blood for serum and peripheral blood mononuclear cells (PBMCs) was collected at multiple timepoints during the vaccination phase (study days 0,7,28,35 and 56) as well as 3,7 and 28-days post-challenge (study days 59,63 and 84). Serum samples were stored at −80±10°C until assayed. PBMCs were isolated on a Ficoll gradient with Leucosep tubes, frozen and stored in liquid nitrogen until used in immunoassays.

### 2.4 Antibodies in lymphocyte secretions (ALS) generation

Frozen PBMCs were thawed, washed and suspended in complete RPMI medium (10% heat inactivated fetal calf serum, 100 U/ml:100 µg/ml penicillin:streptomycin, 2 mM glutamine) at 5 × 10^6^ cells/ml, plated in a sterile 24-well tissue culture plate (1 ml/well) and cultured for 4 days at 37±1 °C with 5% CO_2_. ALS supernatants were collected and frozen at −80±10 °C until used in immunoassays.

### 2.5 Enzyme-linked immunosorbent assay (ELISA)

Serum and ALS samples were assayed by ELISA to determine *S. flexneri* 2a LPS-specific antibody endpoint titres as previously described [21], with the exception of the use of Immulon 1-B ELISA plates (Thermo) and human-specific secondary antibodies (reserve alkaline phosphatase (AP)-conjugated Goat-Anti-Human IgG, IgA or IgM; Seracare; AP-conjugated Mouse-Anti-Human IgG1, IgG2, IgG3 or IgG4; Southern Biotech). Samples that were negative at the starting dilution (the assay limit of detection (LOD)) were assigned a titre corresponding to half of the starting dilution (½ LOD). Immune responders were defined *a priori* as having *a* ≥ 4-fold increase over their baseline titre.

### 2.6 α4β7 PBMC separations

Frozen PBMCs from baseline (day 0), 7 days post-first immunization (day 7), as well as 3 and 7 days post-challenge (days 59 and 63) were thawed, washed and separated into α4β7 positive and negative PBMC populations as previously described using Miltenyi OctoMACS™ columns [22]. The anti-α4β7 monoclonal antibody used for separation (Act-1; NIH AIDS Reagent Program) was conjugated to Alexa Fluor 647, allowing the purity of α4β7 populations to be assessed by flow cytometry. Post-separation, 100 µl from the α4β7+ and α4β7- populations for each volunteer/timepoint were analysed using a FACSCanto II to ensure ≥90% purity in each of the α4β7+ or α4β7- populations (data not shown).

### 2.7 Memory B cell expansion and quality control

Frozen PBMCs from baseline (day 0), 28 days post-second immunization (day 56) and 28 days post-challenge (day 84) were thawed, washed and expanded as previously described [22]. After expansion, the cells were washed twice with mitogen-free complete RPMI medium, adjusted to 5 × 10^6^ cells/ml and cultured as described above to collect ALS from memory B cells. Successful expansion of memory B cell populations was assessed using flow cytometry as previously described [22]. Briefly, criteria for a successful expansion was defined as a post-expansion increase in cells positive for the CD19 B cell marker, as well as *a* ≥ 20% increase in B cells positive for the CD27 memory marker. Additionally, cell viability and cell concentrations were monitored pre- and post-expansion. Samples not meeting the criteria for successful expansion of memory B cell populations were expanded an additional time. If the sample still did not meet the criteria for successful expansion, the sample was excluded from analyses. Additionally, memory B cell responses could only be determined for subjects with a sufficient amount of PBMCs available for the analysis: vaccinees (*n* = 16), placebo recipients (*n* = 27).

### 2.8 Serum bactericidal assay (SBA)

Antibody functionality was assessed by determining *S. flexneri* 2a-specific bactericidal activity as previously described [23]. Serum samples were titrated using serial 3-fold dilutions starting at 1:30 and titres were interpolated from a standard curve using NICE software [24]. A titre of 10, corresponding to one-third of the lowest serum dilution tested, was assigned to samples not exhibiting detectable bactericidal activity at the starting dilution. Immune responders were defined *a priori* as those with *a* ≥ 4-fold increase in bactericidal titre over baseline.

### 2.9 Disease outcomes and definitions

Immune responses pre- and post-challenge in subjects included in the challenge phase of the study (*n* = 30 vaccinees and 29 placebo recipients) were compared across disease outcomes to evaluate the association of immune parameters with progression to disease. The majority of analyses were conducted using the recently developed consensus shigellosis CHIM endpoint (defined as either (1) severe diarrhea OR (2) moderate diarrhea with either fever, ≥1 moderate constitutional/enteric symptom or ≥2 episodes of vomiting in 24 h OR (3) dysentery with either fever, ≥1 moderate constitutional/enteric symptom or ≥2 episodes of vomiting in 24 h) [20]. The following number of subjects progressed to consensus shigellosis post-challenge: 11/30 vaccinees and 17/29 placebo recipients.

In addition, as this consensus definition did not exist prior to study-protocol development, a small subset of immune parameters are also presented using the per protocol *a priori* shigellosis definition (defined as either (1) severe diarrhea OR (2) moderate diarrhea with either fever or ≥1 moderate constitutional/enteric symptom OR (3) dysentery) (Talaat-2020) to compare immune responses across both definitions. The following number of subjects progressed to per-protocol shigellosis post-challenge: 13/30 vaccinees and 17/29 placebo recipients.

### 2.10 Statistics

Normally distributed immune response data (as assessed by distribution plots) were analysed using appropriate parametric tests, otherwise, data were log_10_-transformed prior to analysis. Systemic and memory immune responses across study days were compared back to baseline (day 0) within a given treatment group and shigellosis outcome using a repeated measures ANOVA (RM-ANOVA) with Bonferroni post-hoc test to correct for the multiple comparisons. Missing values (*n* = 4 timepoints) were imputed by carrying the last observation forward. Bonferroni adjusted p-values are reported for all RM-ANOVA tests. Immune responses within a treatment group were compared across shigellosis outcome used using a T-test with comparisons conducted on the peak/maximal immune response either pre- or post-challenge (Table 1).

**Table 1:** LPS-specific systemic and mucosal immune responses either pre- or post-challenge in vaccinated and placebo subjects grouped by consensus shigellosis outcome.

Immune Parameter	Vaccinees (*n* = 30)			Placebos (*n* = 29)		
	No Shigellosis (*n* = 19)	Shigellosis (*n* = 11)	P-Value^b^	No Shigellosis (*n* = 12)	Shigellosis (*n* = 17)	P-Value^b^
Serum IgG						
Pre-Challenge	51,200^a^ (24,879 – 105,367)	9341 (4129 – 21,129)	0.003	2263 (1309 – 3912)	2043 (1089 – 3834)	0.806
Post-Challenge	51,200 (29,018 – 90,340)	8770 (4346 – 17,697)	0.0003	3200 (1881 – 5443)	5220 (3328 – 8187)	0.143
Serum IgA						
Pre-Challenge	3442 (2044 – 5796)	907 (368 – 2240)	0.006	212 (112 – 400)	192 (103 – 358)	0.819
Post-Challenge	2479 (1503 – 4089)	1244 (771 – 2006)	0.062	504 (254 – 1000)	1109 (568 – 2162)	0.097
Serum IgM						
Pre-Challenge	1111 (672 – 1836)	705 (335 – 1486)	0.271	635 (328 – 1228)	452 (240 – 851)	0.443
Post-Challenge	800 (553 – 1157)	662 (353 – 1241)	0.553	898 (550 – 1467)	766 (459 – 1280)	0.643
Serum IgG1						
Pre-Challenge	215 (122 – 379)	69 (44 – 106)	0.006	56 (47 – 67)	57 (44 – 73)	0.963
Post-Challenge	155 (94 – 256)	69 (44 – 106)	0.026	59 (45 – 78)	62 (50 – 78)	0.791
Serum IgG2						
Pre-Challenge	1992 (1050 – 3778)	662 (331 – 1325)	0.025	168 (87 – 323)	226 (123 – 415)	0.491
Post-Challenge	2390 (1308 – 4368)	852 (386 – 1881)	0.035	283 (132 – 606)	617 (395 – 962)	0.053
Pre-Challenge	8076 (5050 – 12,915)	3319 (1223 – 9009)	0.057	884 (447 – 1746)	872 (358 – 2126)	0.982
Post-Challenge	6689 (3703 – 12,084)	4437 (1676 – 11,743)	0.415	1947 (974 – 3893)	4564 (2315 – 8999)	0.078
α4β7+ ALS IgG						
Pre-Challenge (Day 7 Titer)	10 (4 – 25)	2 (1 – 4)	0.010	1 (1 – 2)	1 (1 – 2)	0.542
Post-Challenge	2 (1 – 4)	3 (2 – 6)	0.406	4 (1 – 12)	28 (9 – 89)	0.014
α4β7- ALS IgG						
Pre-Challenge (Day 7 Titer)	13 (5 – 34)	2 (1 – 6)	0.016	1 (1 – 1)	1 (1 – 1)	0.164
Post-Challenge	2 (1 – 3)	1 (1 – 1)	0.010	1 (1 – 2)	3 (2 – 6)	0.008
α4β7+ ALS IgA						
Pre-Challenge (Day 7 Titer)	5 (2 – 11)	2 (1 – 5)	0.128	1 (1 – 1)	1 (1 – 1)	0.799
Post-Challenge	1 (1 – 2)	4 (2 – 11)	0.037	4 (1 – 14)	27 (7 – 99)	0.034
α4β7- ALS IgA						
Pre-Challenge (Day 7 Titer)	3 (1 – 6)	1 (1 – 2)	0.064	1 (1 – 1)	1 (1 – 1)	–
Post-Challenge	1 (1 – 2)	1 (1 – 2)	0.410	1 (1 – 1)	1 (1 – 3)	0.068

Serum IgG3 and serum IgG4 not included due to low/undetectable responses.

Memory B cell IgG and IgA responses due to the low number of subjects/timepoints used in the analysis.

a Geometric Mean Titer (95% Confidence Interval).

b Significance comparing peak/maximal titer either pre- or post-challenge in vaccinated subjects or placebo recipients grouped by consensus shigellosis outcome. P-value determined by T-test of log-transformed titers.

Exploratory analyses were conducted on the association of vaccine efficacy with different ELISA titre cut-points. Exploratory cut-point analyses were performed by plotting receiver operating characteristics (ROC) and investigating sensitivity and specificity across different areas under the curve. Table 2 reports cut-points determined using Liu methodology which defines the optimal cut-point as the maximized product of sensitivity and specificity [25,26]. Table 3 reports cut-points associated with ~70–80% vaccine efficacy. Percent vaccine efficacy is calculated using the shigellosis attack rate (defined as the number of subjects with shigellosis/total number of subjects in that treatment group) as follows: [(attack rate in placebo group – attack rate in vaccinated group)/(attack rate in placebo group)]*100. The sensitivity, specificity, positive predictive value and negative predictive value are listed for each ELISA titre cut-point.

**Table 2:** Liu analysis ELISA titer cut-points across different LPS-specific immune parameters Differentiating vaccinated subjects with consensus shigellosis from vaccinated subjects without consensus shigellosis post-challenge.

Immune Parameterxav	ROC AUC^a^	Optimal Cut-Point^b^	Shigellosis Outcome (n)			Relative Risk^c^ (P-Value)^d^	% Efficacy^e^ (P-Value)^f^	SENS^g^	SPEC^h^	PPV^i^	NPV^j^
			With	Without	Total						
Serum IgG											
Day 28 Titer	0.805	≥12,800	5	16	21	0.357	59.4%	84.2%	54.5%	76.2%	66.7%
		<12,800	6	3	9	(0.042)	(0.021)				
Day 56 Titer	0.792	≥25,600	3	12	15	0.375	65.9%	63.2%	72.7%	80.0%	53.3%
		<25,600	8	7	15	(0.128)	(0.025)				
Max Titer	0.755	≥12,800	7	17	24	0.438	50.2%	89.5%	36.4%	70.8%	66.7%
		<12,800	4	2	6	(0.156)	(0.052)				
Peak Fold Rise	0.842	≥8	4	16	20	0.286	65.9%	84.2%	63.6%	80.0%	70.0%
		<8	7	3	10	(0.015)	(0.009)				
Serum IgA											
Day 28 Titer	0.718	≥800	5	16	21	0.357	59.4%	84.2%	54.5%	76.2%	66.7%
		<800	6	3	9	(0.042)	(0.021)				
Day 56 Titer	0.726	≥800	6	16	22	0.436	53.5%	84.2%	45.5%	72.7%	62.5%
		<800	5	3	8	(0.104)	(0.046)				
Max Titer	0.752	≥1600	4	15	19	0.331	64.1%	78.9%	63.6%	78.9%	63.6%
		<1600	7	4	11	(0.047)	(0.017)				
Peak Fold Rise	0.674	≥8	6	17	23	0.365	55.5%	89.5%	45.5%	73.9%	71.4%
		<8	5	2	7	(0.068)	(0.026)				
Serum IgG1											
Day 28 Titer	0.742	≥100	1	11	12	0.150	85.8%	57.9%	90.9%	91.7%	55.6%
		<100	10	8	18	(0.018)	(0.005)				
Day 56 Titer	0.761	≥100	3	14	17	0.287	69.9%	73.7%	72.7%	82.4%	61.5%
		<100	8	5	13	(0.023)	(0.013)				
Max Titer	0.804	≥100	3	15	18	0.250	71.6%	78.9%	72.7%	83.3%	66.7%
		<100	8	4	12	(0.009)	(0.006)				
Peak Fold Rise	0.796	≥2	3	15	18	0.250	71.6%	78.9%	72.7%	83.3%	66.7%
		<2	8	4	12	(0.009)	(0.006)				
Serum IgG2											
Day 28 Titer	0.691	≥400	8	17	25	0.533	45.4%	89.5%	27.3%	68.0%	60.0%
		<400	3	2	5	(0.327)	0.061				
Day 56 Titer	0.699	≥800	6	17	23	0.365	55.5%	89.5%	45.5%	73.9%	71.4%
		<800	5	2	7	(0.068)	(0.026)				
Max Titer	0.711	≥800	6	17	23	0.365	55.5%	89.5%	45.5%	73.9%	71.4%
		<800	5	2	7	(0.068)	(0.026)				
Peak Fold Rise	0.803	≥4	5	16	21	0.357	59.4%	84.2%	54.5%	76.2%	66.7%
		<4	6	3	9	(0.042)	(0.021)				
Day 28 Titer	0.641	≥3162	5	13	18	0.556	52.6%	68.4%	54.5%	72.2%	50.0%
		<3162	6	6	12	(0.266)	(0.070)				
Day 56 Titer	0.780	≥3415	4	15	19	0.331	64.1%	78.9%	63.6%	78.9%	63.6%
		<3415	7	4	11	(0.047)	(0.017)				
Max Titer	0.708	≥2561	6	17	23	0.365	55.5%	89.5%	45.5%	73.9%	71.4%
		<2561	5	2	7	(0.068)	(0.023)				
Peak Fold Rise	0.763	≥6	5	15	20	0.417	57.4%	78.9%	54.5%	75.0%	60.0%
		<6	6	4	10	(0.108)	(0.040)				
α4β7+ IgG	0.882	≥2	3	17	20	0.150	74.4%	100.0%	66.7%	85.0%	100.0%
		<2	6	0	6	(0.0004)	(0.003)				
α4β7- IgG	0.797	≥2	3	15	18	0.222	71.6%	88.2%	66.7%	83.3%	75.0%
		<2	6	2	8	(0.008)	(0.006)				
α4β7+ IgA	0.696	≥2	3	12	15	0.367	65.9%	70.6%	66.7%	80.0%	54.5%
		<2	6	5	11	(0.103)	(0.025)				
α4β7- IgA	0.647	≥2	2	8	10	0.457	65.9%	47.1%	77.8%	80.0%	43.8%
		<2	7	9	16	(0.399)	(0.065)				
IgG	0.729	≥32	4	10	14	0.653	51.3%	52.6%	63.6%	71.4%	43.8%
		<32	7	9	16	(0.466)	(0.104)				
IgA	0.590	≥64	7	15	22	0.636	45.7%	78.9%	36.4%	68.2%	50.0%
		<64	4	4	8	(0.417)	(0.089)				

a Area under the curve (AUC) of a receiver operator characteristic (ROC) graph.

b Liu cut-point analysis used to determine the optimal point in the AUC which maximizes the product of sensitivity and specificity of the ROC.

c Calculated as: (shigellosis rate among vaccinees at or above the optimal cut-point / shigellosis rate among vaccinees below the optimal cut-point).

d *P*-value determined by Fisher's Exact Test comparing shigellosis rate among vaccinees at or above the optimal cut-point versus shigellosis rate among vaccinees below the optimal cut-point.

e Calculated as: [1 – (shigellosis rate among vaccinees at or above the optimal cut-point / shigellosis rate among placebo recipients)] x 100.

f *P*-value determined by Fisher's Exact Test comparing shigellosis rate among vaccinees at or above the optimal cut-point versus shigellosis rate among placebo recipients.

g Sensitivity = (number of subjects without shigellosis at or above the optimal cut-point / total number of subjects without shigellosis).

h Specificity = (number of subjects with shigellosis at or above the optimal cut-point / total number of subjects with shigellosis).

i Positive Predictive Value = (number of subjects without shigellosis at or above the optimal cut-point / total number of subjects at or above the optimal cut-point).

j Negative Predictive Value = (number of subjects with shigellosis below the optimal cut-point / total number of subjects below the optimal cut-point).

**Table 3:** ELISA titer cut-points across different lps-specific immune parameters associated with 70–80% protection from consensus shigellosis post-challenge.

Immune Parameter	Titer Cut-Point^a^	Shigellosis Outcome (n)			Relative Risk^b^ (P-Value)^c^	% Efficacy^d^ (P-Value)^e^	ROC AUC^f^	SENS^g^	SPEC^h^	PPV^i^	NPV^j^
		With	Without	Total							
Serum IgG											
Day 28 Titer	≥25,600	2	12	14	0.254	75.6%	0.805	63.2%	81.8%	85.7%	56.3%
	<25,600	9	7	16	(0.026)	(0.009)					
Day 56 Titer	≥25,600	3	12	15	0.375	65.9%	0.792	63.2%	72.7%	80.0%	53.3%
	<25,600	8	7	15	(0.128)	(0.025)					
Max Titer	≥51,200	1	9	10	0.200	82.9%	0.755	47.4%	90.9%	90.0%	50.0%
	<51,200	10	10	20	(0.049)	(0.011)					
Peak Fold Rise	≥16	3	14	17	0.287	69.9%	0.842	73.7%	72.7%	82.4%	61.5%
	<16	8	5	13	(0.023)	(0.013)					
Serum IgA											
Day 28 Titer	≥3200	3	13	16	0.328	68.0%	0.718	68.4%	72.7%	81.3%	57.1%
	<3200	8	6	14	(0.057)	(0.013)					
Day 56 Titer	≥3200	2	10	12	0.333	71.6%	0.726	52.6%	81.8%	83.3%	50.0%
	<3200	9	9	18	(0.121)	(0.019)					
Max Titer	≥3200	3	15	18	0.250	71.6%	0.752	78.9%	72.7%	83.3%	66.7%
	<3200	8	4	12	(0.009)	(0.006)					
Peak Fold Rise	≥16	4	15	19	0.331	64.1%	0.674	78.9%	63.6%	78.9%	63.6%
	<16	7	4	11	(0.047)	(0.017)					
Serum IgG1											
Day 28 Titer	≥100	1	11	12	0.150	85.8%	0.742	57.9%	90.9%	91.7%	55.6%
	<100	10	8	18	(0.018)	(0.005)					
Day 56 Titer	≥200	1	10	11	0.173	84.5%	0.761	52.6%	90.9%	90.9%	52.6%
	<200	10	9	19	(0.023)	(0.011)					
Max Titer	≥200	1	12	13	0.131	86.9%	0.804	63.2%	90.9%	92.3%	58.8%
	<200	10	7	17	(0.007)	(0.002)					
Peak Fold Rise	≥4	1	11	12	0.150	85.8%	0.796	57.9%	90.9%	91.7%	55.6%
	<4	10	8	18	(0.018)	(0.005)					
Serum IgG2											
Day 28 Titer	≥1600	3	9	12	0.563	57.4%	0.691	47.4%	72.7%	75.0%	44.4%
	<1600	8	10	18	(0.442)	(0.085)					
Day 56 Titer	≥3200	1	6	7	0.329	75.6%	0.699	31.6%	90.9%	85.7%	43.5%
	<3200	10	13	23	(0.215)	(0.088)					
Max Titer	≥3200	1	6	7	0.329	75.6%	0.711	31.6%	90.9%	85.7%	43.5%
	<3200	10	13	23	(0.215)	(0.088)					
Peak Fold Rise	≥8	2	14	16	0.194	78.7%	0.803	73.7%	81.8%	87.5%	64.3%
	<8	9	5	14	(0.007)	(0.004)					
Day 28 Titer	≥13,806	4	11	15	0.571	54.5%	0.641	57.9%	63.6%	73.3%	46.7%
	<13,806	7	8	15	(0.450)	(0.060)					
Day 56 Titer	≥13,933	1	7	8	0.275	78.7%	0.780	36.8%	90.9%	87.5%	45.5%
	<13,933	10	12	22	(0.199)	(0.042)					
Max Titer	≥15,814	1	7	8	0.275	78.7%	0.708	36.8%	90.9%	87.5%	45.5%
	<15,814	10	12	22	(0.199)	(0.021)					
Peak Fold Rise	≥15	2	9	11	0.384	69.0%	0.763	47.4%	81.8%	81.8%	47.4%
	<15	9	10	19	(0.140)	(0.034)					
α4β7+ IgG	≥4	1	11	12	0.146	85.8%	0.882	64.7%	88.9%	91.7%	57.1%
	<4	8	6	14	(0.014)	(0.005)					
α4β7- IgG	≥2	3	15	18	0.222	71.6%	0.797	88.2%	66.7%	83.3%	75.0%
	<2	6	2	8	(0.008)	(0.006)					
α4β7+ IgA	≥8	1	7	8	0.281	78.7%	0.696	41.2%	88.9%	87.5%	44.4%
	<8	8	10	18	(0.190)	(0.042)					
α4β7- IgA	≥4	1	6	7	0.339	75.6%	0.647	35.3%	88.9%	85.7%	42.1%
	<4	8	11	19	(0.357)	(0.088)					
IgG	≥64	1	8	9	0.233	81.0%	0.729	42.1%	90.9%	88.9%	47.6%
	<64	10	11	21	(0.100)	(0.021)					
IgA	≥64	7	15	22	0.636	45.7%	0.590	78.9%	36.4%	68.2%	50.0%
	<64	4	4	8	(0.417)	(0.089)					

a ELISA titer cut-point chosen based on the titer that provided as close to or between 70 and 80% efficacy.

b Calculated as: (shigellosis rate among vaccinees at or above cut-point / shigellosis rate among vaccinees below cut-point).

c *P*-value determined by Fisher's Exact Test comparing shigellosis rate among vaccinees at or above cut-point versus shigellosis rate among vaccinees below cut-point.

d Calculated as: [1 – (shigellosis rate among vaccinees at or above cut-point / shigellosis rate among placebo recipients)] x 100.

e *P*-value determined by Fisher's Exact Test comparing shigellosis rate among vaccinees at or above cut-point versus shigellosis rate among placebo recipients.

f Area under the curve (AUC) of a receiver operator characteristic (ROC) graph.

g Sensitivity = (number of subjects without shigellosis at or above the cut-point / total number of subjects without shigellosis).

h Specificity = (number of subjects with shigellosis at or above the cut-point / total number of subjects with shigellosis).

i Positive Predictive Value = (number of subjects without shigellosis at or above the cut-point / total number of subjects at or above the cut-point).

j Negative Predictive Value = (number of subjects with shigellosis below the cut-point / total number of subjects below the cut-point).

Spearman correlation of immune response variables was conducted on log-transformed ELISA titres either 7 days post-first immunization (α4β7+ and α4β7- responses) or on day of challenge/day 56 (all other immune parameters). Spearman r values are plotted on a heat map and are also listed with 95% confidence intervals. All statistical tests were conducted in Prism (Version 7 for MAC) and were interpreted in a two-tailed fashion with p-values ≤0.05 considered statistically significant.

### 2.11 Role of funders

The funder of the study (The Wellcome Trust) had no role in the study design data collection, data analysis, data interpretation, or writing of the report. The corresponding author had full access to all the data in the study and had final responsibility for the decision to submit for publication.

## 3 Results

### 3.1 Serum IgG and IgA responses

**Fig. 1:**
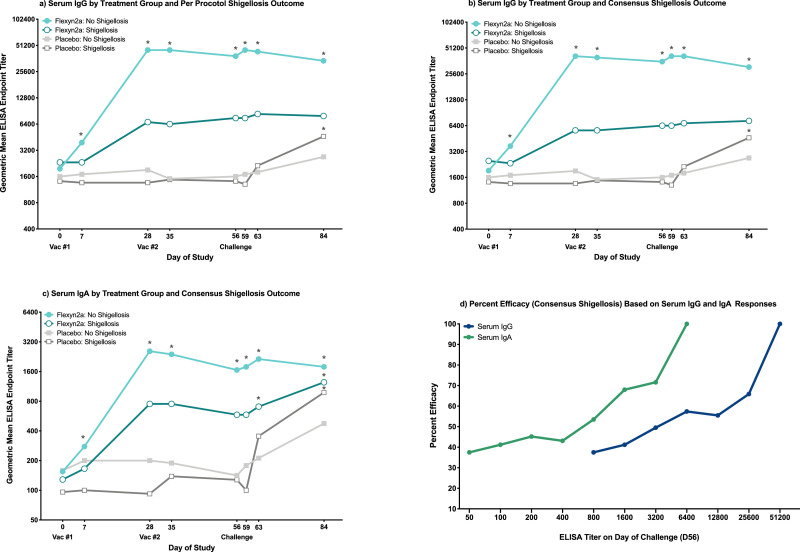
*S. flexneri* 2a LPS-Specific Serum IgG and IgA. (a) *S. flexneri* 2a LPS-specific serum IgG responses grouped by vaccinated subjects with or without per protocol shigellosis, and placebo subjects with or without per protocol shigellosis. (b) *S. flexneri* 2a LPS-specific serum IgG responses grouped by vaccinated subjects with or without consensus shigellosis, and placebo subjects with or without consensus shigellosis. (c) *S. flexneri* 2a LPS-specific serum IgA responses grouped by vaccinated subjects with or without consensus shigellosis, and placebo subjects with or without consensus shigellosis. * = significant difference as compared to baseline titres within the same treatment group/shigellosis outcome. Significance determined by repeated measures ANOVA of log-transformed titres with Bonferroni post-hoc test. (d) Percent efficacy against consensus shigellosis post-challenge in vaccinated subjects across increasing serum IgG and IgA ELISA endpoint titres.

LPS-specific serum IgG and IgA responses and percent seroconversions post-vaccination/challenge have been previously reported by treatment group (Talaat-2020). Vaccinated subjects protected from shigellosis caused by *S. flexneri* 2a (regardless of the definition) had robust increases over baseline serum IgG titres (all comparisons were statistically significant at a level of significance of *p* = 0·05 [RM-ANOVA]; Fig. 1a and b); however, comparable significant increases over baseline were not observed in vaccinated subjects with shigellosis (all *p*>0·05 [RM-ANOVA]; Fig. 1a and b). Similar increases in LPS-specific serum IgA responses over baseline were observed in vaccinated subjects protected from shigellosis (all comparisons were statistically significant at a level of significance of *p* = 0·05 [RM-ANOVA]; Fig. 1c and Supplemental Fig. S1a). Additionally, peak serum IgG and IgA responses prior to challenge were higher in protected vaccinees compared to vaccinees with consensus shigellosis, (serum IgG: *p* = 0·003, serum IgA: *p* = 0·006 [T-Test]; Table 1). A second immunization with Flexyn2a did not increase the serum IgG or IgA responses across all vaccinated subjects, regardless of shigellosis outcome (Fig. 1a–c and Supplemental Fig. S1a). In placebo recipients, no differences were observed in peak serum IgG or IgA titres post-challenge across consensus shigellosis outcome (Table 1).

Oral challenge with *S. flexneri* 2a, 2457T did not significantly increase the LPS-specific serum IgG responses in vaccinated subjects, regardless of shigellosis outcome (all *p*>0·999 [RM-ANOVA]; Fig. 1a and b). Interestingly, while not statistically significant, serum IgA responses in unprotected vaccinees increased post-challenge (all *p*>0·1 [RM-ANOVA]; Fig. 1c), a trend not observed in protected vaccinees. Significant increases over baseline LPS-specific serum IgG and IgA titres were observed post-challenge in placebo recipients progressing to consensus shigellosis (all comparisons were statistically significant at a level of significance of *p* = 0·005 [RM-ANOVA]; Fig. 1b and c). Similar magnitudes of increase in serum IgG or IgA responses were not observed in placebo recipients without shigellosis.

Serum IgG and IgA titres on day of challenge predicted efficacy post-challenge. Using Liu cut-point analyses (Table 2), a serum IgG titre of 25,600 provided 66% efficacy while a lower serum IgA titre of 800 provided 54% efficacy in vaccinated subjects (Table 2). Percent efficacy rose sharply thereafter with increasing serum IgG or IgA titres (Fig. 1d and Table 3).

### 3.2 Serum IgG subclass and IgM responses

**Fig. 2:**
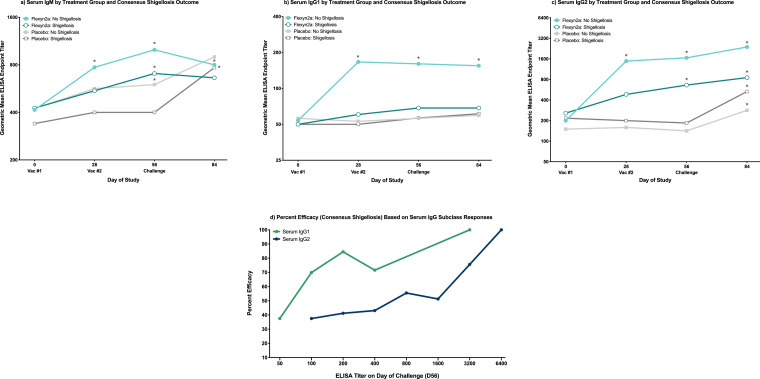
*S. flexneri* 2a LPS-Specific Serum IgM, IgG1 and IgG2. *S. flexneri* 2a LPS-specific serum IgM (a), IgG1 (b) and IgG2 (c) geometric mean ELISA endpoint titres grouped by vaccinated subjects with or without consensus shigellosis, and placebo subjects with or without consensus shigellosis. * = significant difference as compared to baseline titres within the same treatment group/shigellosis outcome. Significance determined by repeated measures ANOVA of log-transformed titres with Bonferroni post-hoc test. (d) Percent efficacy against consensus shigellosis post-challenge in vaccinated subjects across increasing serum IgG1 and IgG2 ELISA endpoint titres measured at time of challenge (day 56).

Immunization with Flexyn2a induced significant increases over baseline LPS-specific serum IgG1 responses (all comparisons were statistically significant at a level of significance of *p* = 0·005 [RM-ANOVA]; Supplemental Fig. S2b). When segregated by consensus shigellosis outcome, there is a clear differentiation between protected and unprotected vaccinees in the serum IgG1 increases over baseline (all comparisons were statistically significant at a level of significance of *p* = 0·01 [RM-ANOVA]; Fig. 2b). Vaccinated subjects protected from shigellosis caused by *S. flexneri* 2a had significantly higher peak serum IgG1 responses compared to vaccinees with shigellosis, both pre-challenge (*p* = 0·006 [T-Test]; Table 1) as well as post-challenge (*p* = 0·026 [T-Test]; Table 1). Furthermore, serum IgG1 titres in unprotected vaccinees and all placebo recipients were similar in magnitude throughout the observation period (Fig. 2b). Nearly identical responses were observed with serum IgG1 across the per protocol shigellosis definition (Supplemental Fig. S1b). Serum IgG1 titres on day of challenge were also associated with vaccine efficacy with a titre as low as 100 providing 70% efficacy in vaccinated subjects (Fig. 2d and Table 2) which increased sharply to 85% when a titre of 200 was reached (Fig. 2d and Table 3).

LPS-specific serum IgG2 responses showed a different trend compared to serum IgG1 responses with all vaccinated subjects having detectable increases in serum IgG2 responses, regardless of shigellosis outcome (Fig. 2c and Supplemental Figs. S1c and S2b). Although pre-challenge peak serum IgG2 titres were higher in protected vaccinees than in vaccinees with shigellosis (*p* = 0·025 [T-Test]; Table 1), it was at a lower level compared to IgG1. Placebo recipients had increased LPS-specific serum IgG2 responses over baseline by day 84, regardless of shigellosis outcome (all comparisons were statistically significant at a level of significance of *p* = 0·01 [RM-ANOVA]; Fig. 2c). Similar responses were observed when comparing serum IgG2 titres across per protocol shigellosis (Supplemental Fig. S1c). Serum IgG2 titres at time of challenge (day 56) were associated with percent efficacy in vaccinated subjects however, at a higher magnitude of response as compared to serum IgG1. A serum IgG2 titre of 800 provided 56% efficacy (Fig. 2d and Table 2) while a 4-fold increase in serum IgG2 titre to 3200 provided 76% efficacy (Fig. 2d and Table 3).

Immunization with Flexyn2a induced low but significant LPS-specific serum IgM titres 28 days post-first immunization (*p* = 0·002 [RM-ANOVA]; Supplemental Fig. S2a). When vaccinated subjects are further grouped by shigellosis outcome, the significant increase in serum IgM on day 28 only remains for protected vaccinees (*p* = 0·014 [RM-ANOVA]; Fig. 2a). No differences were observed in peak serum IgM titres across vaccinated subjects with or without shigellosis either pre- or post-challenge (Table 1). Minimal to undetectable increases in LPS-specific serum IgG3 and IgG4 responses were observed throughout the study with no significant differences at any time point or across shigellosis outcome within treatment group (Supplemental Fig. S2c).

### 3.3 Bactericidal responses

**Fig. 3:**
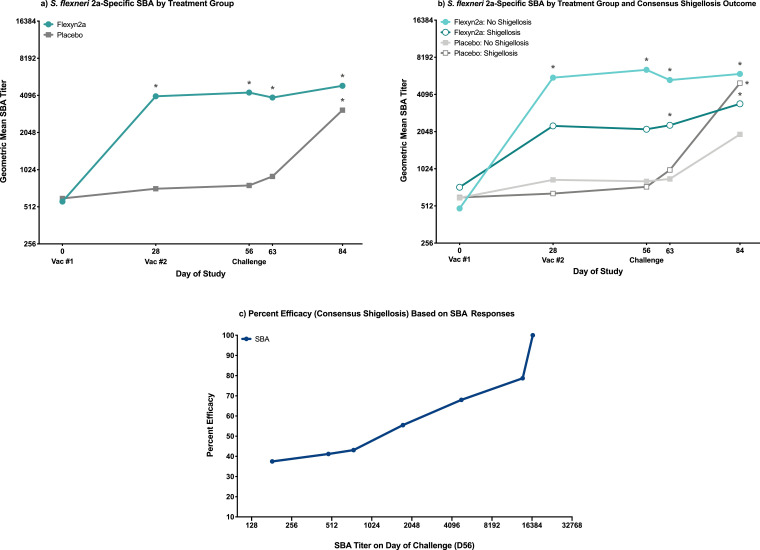
Bactericidal responses. (a) *S. flexneri* 2a-specific geometric mean SBA endpoint titres grouped by vaccinated or placebo subjects. * = significant difference as compared to baseline titres within treatment group. Significance determined by repeated measures ANOVA of log-transformed titres with Bonferroni post-hoc test. (b) *S. flexneri* 2a-specific geometric mean SBA endpoint titres grouped by vaccinated subjects with or without consensus shigellosis, and placebo subjects with or without consensus shigellosis. * = significant difference as compared to baseline titres within the same treatment group/shigellosis outcome. Significance determined by repeated measures ANOVA of log-transformed titres with Bonferroni post-hoc test. (c) Percent efficacy against consensus shigellosis post-challenge in vaccinated subjects across increasing serum bactericidal titres measured at time of challenge (day 56).

Flexyn2a induced robust *S. flexneri* 2a-specific bactericidal activity after one immunization, with responses in the vaccinated group remaining elevated over baseline through study day 84 (all *p* ≤ 0·0001 [RM-ANOVA]; Fig. 3a). While vaccinees protected from shigellosis caused by *S. flexneri* 2a had higher SBA titres compared to unprotected vaccinees, this difference was not statistically significant (Fig. 3b and Table 1). Across all vaccinated subjects, neither a second immunization with Flexyn2a, nor oral challenge with *S. flexneri* 2a, 2457T increased the overall magnitude of the bactericidal responses (Fig. 3a); however, a second immunization with Flexyn2a did increase the frequency of responders from 70 to 83% (data not shown). Placebo recipients progressing to shigellosis showed a similar magnitude of increase in bactericidal responses by day 84 (*p* = 0.0006 [RM-ANOVA]; Fig. 3b) with responses in these individuals surpassing those observed in unprotected vaccinees. The relationship between percent efficacy and bactericidal titre increased steadily with an SBA titre of 3415 providing 64% efficacy (Fig. 3c and Table 2). When the bactericidal titre is increased to 13,933, the percent efficacy also increases to 79% (Fig. 3c and Table 3).

### 3.4 α4β7 ALS antibody responses

**Fig. 4:**
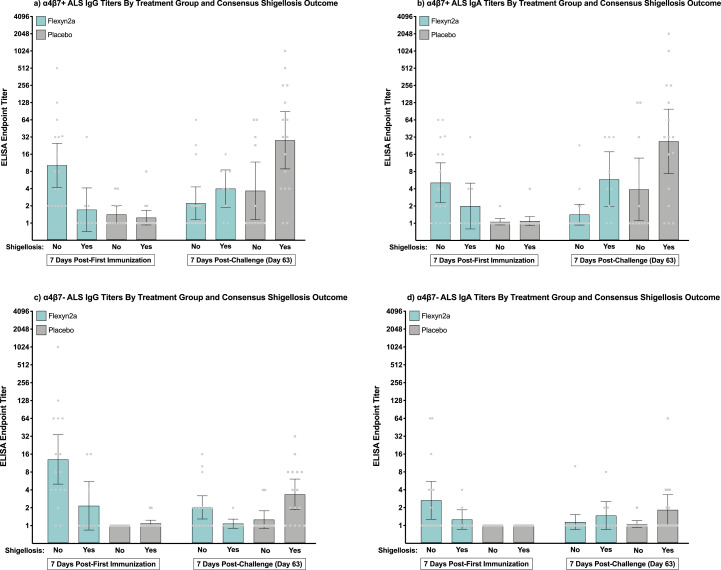
α4β7+ and α4β7- ALS IgG and IgA Responses. Individual *S. flexneri* 2a LPS-specific α4β7+ and α4β7- ALS IgG and IgA ELISA endpoint titres with group geometric mean and 95% confidence intervals either 7 days post-first immunization or 7 days post-challenge (day 63) grouped by treatment and consensus shigellosis outcome. (a) α4β7+ ALS IgG; (b) α4β7+ ALS IgA; (c) α4β7- ALS IgG; (d) α4β7- ALS IgA.

Flexyn2a induced moderate to robust increases in both α4β7+ and α4β7- ALS antibody responses 7 days post-first immunization (Supplemental Fig. S3). Protected vaccinees had higher α4β7+ ALS IgG responses on day 7 compared to unprotected vaccinees (*p* = 0·01 [T-Test]; Table 1). Additionally, α4β7+ ALS IgG responses in protected vaccinees were higher 7 days post-first immunization compared to 7 days post-oral challenge with *S. flexneri* 2a, 2457T (Fig. 4a). In unprotected vaccinees a minimal boost in α4β7+ ALS IgG responses from day 7 to day 63 was observed (Fig. 4a). Flexyn2a also increased the α4β7+ ALS IgA responses post-first vaccination; however, there were no differences between protected and unprotected vaccinated subjects (Table 1 and Fig. 4b). The α4β7+ ALS IgA responses post-challenge showed similar trends to the α4β7+ ALS IgG responses, with protected vaccinees having a higher magnitude of α4β7+ ALS IgA responses on day 7 compared to day 63 while oral challenge increased the α4β7+ ALS IgA responses in unprotected vaccinees (Fig. 4b).

Vaccinated subjects protected from shigellosis caused by *S. flexneri* 2a had significant increases in α4β7- ALS IgG responses 7 days post-first immunization as compared to unprotected vaccinees (*p* = 0·016 [T-Test]; Table 1 and Fig. 4c); however, a similar magnitude of increase in α4β7- ALS IgA responses was not observed (Table 1 and Fig. 4d). Both α4β7+ and α4β7- antibody titres were associated with percent efficacy with any increase (≥2-fold rise over baseline) in α4β7+ or α4β7- antibody titre associated with 60–70% efficacy (Supplemental Fig. S4 and Tables 2 and 3). LPS-specific α4β7+ IgG responses quickly reached 86% efficacy at a low titre of 4 while the α4β7- IgG and α4β7 IgA responses did not reach this same level of efficacy until a titre of between 8 and 32 was reached (Supplemental Fig. S4 and Tables 2 and 3).

### 3.5 Memory B cell responses

**Fig. 5:**
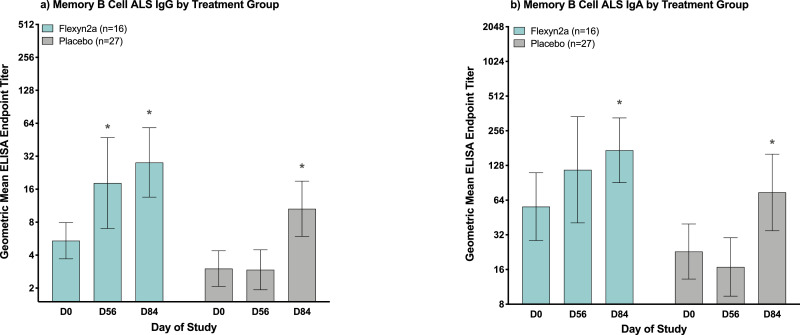
Memory B cell responses. *S. flexneri* 2a LPS-specific memory B cell ALS IgG (a) and IgA (b) geometric mean ELISA endpoint titres with 95% confidence intervals at baseline (day 0), day of challenge (day 56) and 28 days post-challenge (day 84), grouped by vaccinated or placebo subjects. * = significant difference as compared to baseline titres within treatment group as determined by repeated measures ANOVA of log-transformed titres with Bonferroni post-hoc test.

Immunization with Flexyn2a induced significant increases in LPS-specific memory B cell IgG responses by day 56 (*p* = 0·05 [RM-ANOVA]; Fig. 5a) which further increased post-challenge. Memory B cell IgG responses in vaccinated subjects on day of challenge (day 56) were comparable to the memory B cell IgG responses detected in placebo recipients post-challenge (day 84) (Fig. 5a). Flexyn2a also increased the LPS-specific memory B cell IgA responses post-immunization; however, increases over baseline were not significant until after subjects were challenged with *S. flexneri* 2a, 2457T (*p* = 0·004 [RM-ANOVA]; Fig. 5b).

### 3.6 Correlation of immune parameters in vaccinated subjects

**Fig. 6:**
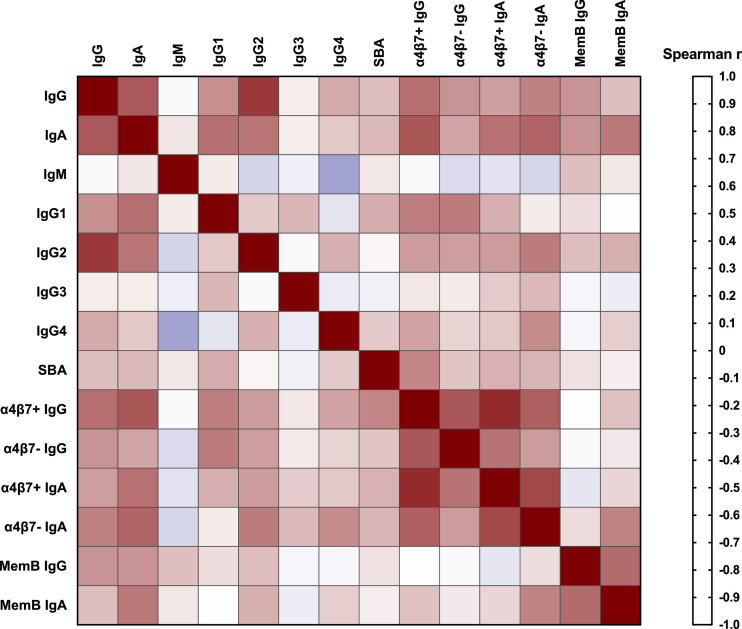
Spearman correlation of immune parameters in vaccinated subjects. Spearman correlation heat map of immune parameters in vaccinated subjects either 7 days post-first immunization (α4β7+ and α4β7- responses) or on day of challenge/day 56 (all other immune parameters).

Not surprisingly, the strongest correlation in immune parameters among vaccinated subjects was between α4β7+ IgG and IgA responses (Spearman *r* = 0·87; Fig. 6) while α4β7- IgG and IgA were correlated at a lower level (Spearman *r* = 0·51; Fig. 6). With the exception of the α4β7- IgG responses, which were most strongly correlated with serum IgG1 (Spearman *r* = 0·61; Fig. 6), all other α4β7+ and α4β7- responses showed a moderate correlation with serum IgA (Spearman *r* = 0·64–0·73; Fig. 6). Serum IgA also correlated with serum IgG1 (Spearman *r* = 0·65; Fig. 6) and IgG2 (Spearman *r* = 0·63; Fig. 6); however, the immune parameters with the strongest serum IgA correlation were α4β7+ IgG (Spearman *r* = 0·73; Fig. 6) and serum IgG (Spearman *r* = 0·72; Fig. 6). Of the serum IgG subclass responses, IgG2 was most strongly correlated with serum IgG (Spearman *r* = 0·82; Fig. 6) whereas serum IgG1 was most highly correlated with serum IgA followed by α4β7+ IgG and α4β7- IgG responses (both Spearman *r* = 0·61; Fig. 6). Bactericidal activity had the strongest correlation with α4β7+ IgG responses (Spearman *r* = 0·58; Fig. 6) followed by serum IgG1 responses (Spearman *r* = 0·46; Fig. 6). Memory B cell IgG and IgA responses correlated best with each other (Spearman *r* = 0·66; Fig. 6) however, memory B cell IgG also correlated with serum IgG and IgA responses (Spearman *r* = 0·53–0·54; Fig. 6). Aside from the correlation with memory B cell IgG responses, memory B cell IgA responses correlated best with serum IgA responses (Spearman *r* = 0·62; Fig. 6), as well as α4β7- IgA responses (Spearman *r* = 0·59; Fig. 6). All spearman *r* values, and 95% confidence intervals are listed in Supplemental Table S1.

## 4 Discussion

Immunization with one dose of Flexyn2a elicited robust LPS-specific serum IgG and IgA antibody responses, as well as functional bactericidal antibody responses. Flexyn2a also induced low, but detectable, LPS-specific serum IgM responses 28 days post-first immunization; however, as IgM responses typically peak within 7–14 days post-first antigenic exposure, it is possible that the full magnitude of serum IgM responses was not captured as earlier timepoints were not included in the current analyses. Interestingly, while Flexyn2a induced both LPS-specific serum IgG1 and IgG2 responses, serum IgG1 responses were more closely correlated with protection from shigellosis caused by *S. flexneri* 2a as compared to serum IgG2, regardless of the higher magnitude of serum IgG2 titres observed post-vaccination. Additionally, an increase in serum IgG2 levels post-challenge was observed in placebo recipients however, no increases in serum IgG1 responses were observed in the placebo group post-challenge, indicating that oral challenge induces a different response with respect to serum IgG subclasses as compared to parenteral immunization with Flexyn2a.

When considering overall magnitude of the response, the IgG subclass titres observed in this study confirm previously documented differences across IgG1 and IgG2 responses based on *Shigella* serotype, with *S. flexneri* 2a and *S. sonnei* inducing either an IgG2 or IgG1 dominated response, respectively [22,27,28]. However, in the current study, when the focus in placed on the association of each IgG subclass with protection from shigellosis rather than overall magnitude of response, a potentially important role for *Shigella*-specific serum IgG1 in protection from shigellosis post-parenteral immunization is suggested. As IgG1 antibodies are highly efficient at activating complement as compared to IgG2 [29], they may be contributing to the killing of shigellae at a higher level while in the intestinal lamina propria during the process of transudation. The increased complement activation efficiency of IgG1 antibodies may therefore require a lower magnitude or threshold of response for them to be effective. Additionally, the role of IgG1 antibodies may be especially important in the context of the α4β7+ ALS IgG responses observed in this study (see below), which is further evidenced by the correlation between these two parameters.

Indeed, another important finding in this study is the induction post-vaccination of *Shigella* LPS-specific α4β7+ IgG and IgA secreting B cells which are likely homing to the gut. A traditionally accepted paradigm in regard to parenterally delivered vaccines in the context of mucosal pathogens is their inability to induce mucosal immune responses, making these vaccines reliant on robust systemic responses leading to transudation of systemic antibodies into mucosal effector sites [[30], [31], [32]]. While this paradigm has been previously challenged in the context of other pathogen-specific parenterally delivered vaccines such as influenza, polio and tetanus [[33], [34], [35]], mucosal immune responses observed in these, and similar, studies may be a result of a secondary antigenic exposure after an initial oral priming exposure [30,36]. Furthermore, investigations of mucosal immune responses have largely focused on the importance of IgA secreting gut-homing B cells or secretory IgA in mucosal secretions [32,37,38]. While there is no doubt that secretory IgA plays a vital role in protection against mucosal pathogens [39], investigations into the protective capacity of antigen-specific IgG secreting α4β7+ *B* cells against mucosal pathogens have been limited.

Parenteral immunization with the Flexyn2a bioconjugate vaccine induced robust LPS-specific α4β7+ IgG ALS responses in vaccinated subjects protected from shigellosis caused by *S. flexneri* 2a. While lower in magnitude, vaccinees also had increased α4β7+ IgA ALS titres, demonstrating the ability of Flexyn2a to induce both an IgG and IgA α4β7+ immune response. To our knowledge, this is the first study to report antigen-specific B cells positive for the gut-homing marker post-parenteral immunization with conjugate vaccine for an enteric pathogen. The induction of α4β7+ IgG and IgA secreting B cells post-immunization may offer a mechanistic explanation of the protection afforded by the bioconjugate vaccine as LPS-specific B cells may home to the gut and produce antibodies at the site of infection, which can be actively secreted (IgA) or passively transudated (IgG) into the lumen. This finding could be especially important in the context of the α4β7+ LPS-specific IgG secreting B cells as these antibodies may contribute to the neutralization, or direct killing (either via complement activation or opsonization) of shigellae in the lamina propria that have transcytosed across the intestinal epithelial barrier. Of course, the immune responses induced after natural infection or pre-existing immunity may offer different mechanisms of protection.

Similar investigations into the LPS-specific α4β7+ IgG and IgA responses were conducted on samples from the Phase 1 clinical study of Flexyn2a administered with and without Alum [18]. Results from the Phase 1 α4β7 analyses (unpublished data) mirrored the responses achieved in the current study, further confirming the ability of Flexyn2a to induce an antigen-specific α4β7+ antibody response. Although the addition of Alum did not influence the magnitude of the LPS-specific α4β7+ IgG and IgA response or the number of responders, it is important to consider the possibility that alternative adjuvants with enhanced capacity to augment mucosal immune responses or influence the phenotype of the immune response may provide different results, warranting further investigations [40]. Additionally, as recently recommended [41], future studies should investigate the IgG subclass responses as well as the bactericidal activity in α4β7+ ALS samples. Determining the dominant subclass of the α4β7+ IgG secreting B cells and the bactericidal ability of these antibodies could further inform mechanisms of protection during *Shigella* infection.

A second immunization with Flexyn2a or oral challenge with *S. flexneri* 2a, 2457T did not substantially boost any of the tested immune responses in protected vaccinees. In contrast, challenge with *S. flexneri* 2a, 2457T increased multiple *Shigella*-specific immune responses in unprotected vaccinees as well as placebo subjects, specifically in serum IgA, serum IgG2 and α4β7+ IgA responses. The post-challenge immune response dampening in protected vaccinees may be due to a reduced duration of antigenic exposure during the challenge phase suggesting that, in some cases, immunization with Flexyn2a is capable of reducing the number of shigellae that are able to invade the mucosal epithelium. Additionally, antibodies in the lamina propria may also work to reduce the number of shigellae that are able to invade the basolateral surface of intestinal epithelial cells. Interestingly, this phenomenon of an immune response dampening has also been observed in other enteric human challenge studies [42,43].

Although subjects meeting multiple exclusion criteria, including serological evidence of prior exposure to *S. flexneri* 2a were excluded from the study, only 59 and 62% of the placebo recipients met the consensus and per protocol shigellosis endpoint, respectively, similar to previous reports [22,44,45]. Prior *S. sonnei* and ETEC CHIMs have identified increased serum IgA titres to key antigens at baseline as predictive of post-challenge disease risk [22,44]. Similar investigations were performed in the current study however no differences were observed in the pre-challenge serum IgA responses of placebo subjects across shigellosis outcome. Interestingly, when additional baseline immune parameters were investigated, placebo subjects not progressing to shigellosis were found to have significantly higher LPS-specific memory B cell IgA responses on the day of challenge as compared to placebo subjects with shigellosis (*p* = 0·035 [T-Test of log-transformed titres]; data not shown). This difference in LPS-specific memory B cell IgA response prior to challenge has also been recently reported in a *S. sonnei* CHIM [22]. While no other differences in immune responses on day of challenge were observed, these data, as well as data from other CHIMs, demonstrate the importance of additional investigations into reduced disease risk post-challenge as this may have a substantial effect in the assessment of vaccine efficacy.

Consistent with results from the Phase 1 study of Flexyn2a [18], and other clinical assessments of *Shigella* conjugate vaccines [15,16], parenteral immunization with the bioconjugate induced robust systemic immune responses. Flexyn2a also induced functional antibody responses as well as LPS-specific memory and α4β7+ *B* cells. This study also confirmed the vaccine's previously reported excellent safety profile and demonstrated the vaccine's ability to induce a protective immune response in a *S. flexneri* 2a CHIM setting (Talaat-2020) [17,18]. The combination of a robust safety and immunogenicity profile, with efficacy against oral challenge and a simple, affordable and reproducible manufacturing process [19,46] supported the cGMP production of a quadrivalent *Shigella* bioconjugate vaccine formulation. The quadrivalent formulation is currently undergoing safety and immunogenicity evaluations in an age-descending trial in Kenya (ClinicalTrials.gov Identifier: NCT04056117). Promising results achieved in the age-descending trial may support pivotal field trials of the final vaccine construct.

## Declaration of Competing Interest

CA, ALB, JB, KAC, BD, AMD, DE, BF, RF, VGF, JH, RWK, PM, DS, KRT, HPW report grants from Wellcome Trust during the conduct of the study. SC, CKP, MSR report no conflicts of interest for any of the materials presented in the manuscript.

## Appendix Supplementary materials

Image, application 1
