# Supplementary material for: Immune response characterization in a human challenge study with a Shigella flexneri 2a bioconjugate vaccine
Source: eBioMedicine. 2021 Apr 1;66:103308. doi: 10.1016/j.ebiom.2021.103308 (PMC8047506; doi:10.1016/j.ebiom.2021.103308)
Supplement: Supplementary file 1 [file mmc1.docx]

**Supplemental Figure S1. Serum IgA and IgG Subclass Responses by Treatment Group and Per Protocol Shigellosis.** *S. flexneri* 2a LPS-specific serum IgA (a), IgG1 (b) and IgG2 (c) geometric mean ELISA endpoint titres grouped by vaccinated subjects with or without per protocol shigellosis, and placebo subjects with or without per protocol shigellosis. * = significant difference as compared to baseline titres within the same treatment group/shigellosis outcome. Significance determined by repeated measures ANOVA of log-transformed titres with Bonferroni post-hoc test.

**Supplemental Figure S2. Serum IgM and IgG Subclass Responses by Treatment Group.** *S. flexneri* 2a LPS-specific serum IgM (a), IgG1 and IgG2 (b), IgG3 and IgG4 (c) geometric mean ELISA endpoint titres grouped by vaccinated or placebo subjects. * = significant difference as compared to baseline titres within the same treatment group/shigellosis outcome. Significance determined by repeated measures ANOVA of log-transformed titres with Bonferroni post-hoc test.

**Supplemental Figure S3. α4β7+ and α4β7- ALS IgG and IgA Responses Over Time.** *S. flexneri* 2a LPS-specific ALS IgG (a) and IgA (b) geometric mean α4β7+ (solid lines) and α4β7- (dashed lines) ALS ELISA endpoint titres at baseline (day 0), 7 days post-first immunization (day 7) and 7 days post-challenge (day 63), grouped by treatment.

**Supplemental Figure S4. Percent Efficacy in Vaccinees Across Multiple α4β7** **ALS Titre Cut-Offs.** Percent efficacy against consensus shigellosis post-challenge in vaccinated subjects across increasing α4β7+ and α4β7- antibody titres.

| **Supplemental Table S1. Spearman Correlation Matrix of Immune Response Parameters in Vaccinated Subjects^a^** | | | | | | | | | | | | | |
| --- | --- | --- | --- | --- | --- | --- | --- | --- | --- | --- | --- | --- | --- |
|  |  |  |  |  |  |  |  |  |  |  |  |  |  |
|  | **IgG** | **IgA** | **IgM** | **IgG1** | **IgG2** | **IgG3** | **IgG4** | **SBA** | **α4β7+ IgG** | **α4β7- IgG** | **α4β7+ IgA** | **α4β7- IgA** | **MemB IgG** |
| **IgG** | 1.00  (N/A) |  |  |  |  |  |  |  |  |  |  |  |  |
| **IgA** | **0.72**  **(0.48 to 0.86)^b^** | 1.00  (N/A) |  |  |  |  |  |  |  |  |  |  |  |
| **IgM** | 0.18  (-0.21 to 0.51) | 0.28  (-0.10 to 0.59) | 1.00  (N/A) |  |  |  |  |  |  |  |  |  |  |
| **IgG1** | **0.55**  **(0.22 to 0.76)** | **0.65**  **(0.37 to 0.82)** | 0.26  (-0.12 to 0.57) | 1.00  (N/A) |  |  |  |  |  |  |  |  |  |
| **IgG2** | **0.82**  **(0.65 to 0.91)** | **0.63**  **(0.34 to 0.81)** | 0.00  (-0.37 to 0.37) | **0.37**  **(-0.004 to 0.65)** | 1.00  (N/A) |  |  |  |  |  |  |  |  |
| **IgG3** | 0.25  (-0.13 to 0.57) | 0.26  (-0.13 to 0.57) | 0.13  (-0.25 to 0.48) | **0.43**  **(0.07 to 0.69)** | 0.17  (-0.21 to 0.51) | 1.00  (N/A) |  |  |  |  |  |  |  |
| **IgG4** | **0.46**  **(0.11 to 0.71)** | **0.37**  **(-0.001 to 0.65)** | -0.23  (-0.55 to 0.15) | 0.07  (-0.31 to 0.43) | **0.44**  **(0.086 to 0.70)** | 0.11  (-0.27 to 0.46) | 1.00  (N/A) |  |  |  |  |  |  |
| **SBA** | **0.40**  **(0.04 to 0.67)** | **0.42**  **(0.06 to 0.68)** | 0.27  (-0.11 to 0.58) | **0.46**  **(0.10 to 0.71)** | 0.23  (-0.16 to 0.55) | 0.13  (-0.25 to 0.48) | **0.37**  **(-0.004 to 0.65)** | 1.00  (N/A) |  |  |  |  |  |
| **α4β7+ IgG** | **0.65**  **(0.34 to 0.83)** | **0.73**  **(0.46 to 0.87)** | 0.17  (-0.24 to 0.53) | **0.61**  **(0.27 to 0.81)** | **0.50**  **(0.13 to 0.75)** | 0.27  (-0.14 to 0.61) | **0.49**  **(0.11 to 0.74)** | **0.58**  **(0.23 to 0.79)** | 1.00  (N/A) |  |  |  |  |
| **α4β7- IgG** | **0.53**  **(0.17 to 0.77)** | **0.48**  **(0.10 to 0.74)** | 0.03  (-0.37 to 0.42) | **0.61**  **(0.28 to 0.81)** | **0.50**  **(0.13 to 0.75)** | 0.26  (-0.15 to 0.60) | 0.33  (-0.07 to 0.65) | 0.38  (-0.02 to 0.67) | **0.72**  **(0.46 to 0.87)** | 1.00  (N/A) |  |  |  |
| **α4β7+ IgA** | **0.50**  **(0.12 to 0.75)** | **0.64**  **(0.33 to 0.83)** | 0.07  (-0.34 to 0.45) | **0.45**  **(0.06 to 0.72)** | **0.51**  **(0.14 to 0.76)** | 0.36  (-0.04 to 0.66) | 0.37  (-0.03 to 0.67) | **0.44**  **(0.05 to 0.71)** | **0.87**  **(0.71 to 0.94)** | **0.64**  **(0.32 to 0.82)** | 1.00  (N/A) |  |  |
| **α4β7- IgA** | **0.59**  **(0.25 to 0.80)** | **0.68**  **(0.39 to 0.85)** | 0.01  (-0.39 to 0.40) | 0.26  (-0.16 to 0.59) | **0.61**  **(0.28 to 0.81)** | **0.42**  **(0.03 to 0.70)** | **0.56**  **(0.20 to 0.78)** | **0.43**  **(0.04 to 0.71)** | **0.70**  **(0.42 to 0.86)** | **0.51**  **(0.14 to 0.75)** | **0.77**  **(0.54 to 0.89)** | 1.00  (N/A) |  |
| **MemB IgG** | **0.53**  **(0.21 to 0.76)** | **0.54**  **(0.21 to 0.76)** | **0.40**  **(0.04 to 0.67)** | 0.30  (-0.08 to 0.60) | **0.40**  **(0.04 to 0.67)** | 0.16  (-0.22 to 0.50) | 0.16  (-0.22 to 0.50) | 0.29  (-0.09 to 0.60) | 0.19  (-0.22 to 0.55) | 0.18  (-0.24 to 0.54) | 0.08  (-0.32 to 0.47) | 0.31  (-0.10 to 0.63) | 1.00  (N/A) |
| **MemB IgA** | **0.40**  **(0.03 to 0.67)** | **0.62**  **(0.32 to 0.81)** | 0.28  (-0.11 to 0.59) | 0.20  (-0.18 to 0.53) | **0.45**  **(0.09 to 0.70)** | 0.12  (-0.26 to 0.47) | 0.35  (-0.03 to 0.64) | 0.25  (-0.13 to 0.57) | **0.39**  **(-0.01 to 0.68)** | 0.27  (-0.15 to 0.60) | 0.33  (-0.08 to 0.64) | **0.59**  **(0.25 to 0.80)** | **0.66**  **(0.39 to 0.83)** |
|  | | | | | | | | | | | | | |
| ^a^ Spearman correlation of log-transformed ELISA titers either 7 days post-first immunization (α4β7+ and α4β7- responses) or on day of challenge/day 56 (all other immune parameters).  ^b^ Spearman r values with 95% confidence intervals in parentheses. Bolded values represent significant correlations with p-values ≤0.05. | | | | | | | | | | | | | |
